# Simultaneous bilateral total knee replacement in a patient with achondroplasia and severe varus deformity: a case report

**DOI:** 10.1093/jscr/rjaf855

**Published:** 2025-10-24

**Authors:** Danil Chernov, Nicholas Frappa, Thomas Listopadzki, Matthew Alben, Alexander Kovacs, Rajkumar Selvanayagam, Sridhar R Rachala

**Affiliations:** Jacobs School of Medicine and Biomedical Sciences, 955 Main St. Buffalo, NY 14203, United States; Jacobs School of Medicine and Biomedical Sciences, 955 Main St. Buffalo, NY 14203, United States; Department of Orthopaedics and Sports Medicine, 462 Grider St. Buffalo, NY 14215, United States; Department of Orthopaedics and Sports Medicine, 462 Grider St. Buffalo, NY 14215, United States; Department of Orthopaedics and Sports Medicine, 462 Grider St. Buffalo, NY 14215, United States; Department of Orthopaedics and Sports Medicine, 462 Grider St. Buffalo, NY 14215, United States; Department of Orthopaedics and Sports Medicine, 462 Grider St. Buffalo, NY 14215, United States

**Keywords:** achondroplasia, total knee arthroplasty, bilateral, simultaneous, osteoarthritis

## Abstract

We present a unique case of a 61-year-old female with achondroplasia and end-stage osteoarthritis of both knees who presented with severe pain and bilateral varus deformities of over 40 degrees. The patient underwent single-stage bilateral total knee arthroplasty (TKA) using cemented rotating-hinge implants with long stems. At 5-year follow-up, the patient has neutral limb alignment, pain-free ambulation without assistive devices, and stable implants on radiographs. This is one of the first reported cases of simultaneous bilateral TKA in a patient with achondroplasia and extreme bilateral varus deformity, highlighting how successful correction can be achieved using hinged implants and careful surgical planning, underscoring the feasibility of one-stage bilateral intervention in this rare and challenging scenario.

## Introduction

Achondroplasia is the most common skeletal dysplasia (SD) caused by an activating fibroblast growth factor receptor-3 mutation (FGFR-3), with a prevalence of 1 in 20 000–30 000 live births [1]. Abnormal endochondral ossification leads to shortened proximal long bones and characteristic genu varum [2]. Osteoarthritis (OA) in this population presents unique challenges due to anatomic and biomechanical alterations [3].

Total knee arthroplasty (TKA) is indicated for severe deformity and pain, though the exact incidence is generally unknown [4]. Moore *et al*. identified only 285 TKA cases in patients with achondroplasia over a decade [5]. Stancil *et al*. [6] described staged bilateral TKAs with hinged components, and Walter *et al*. reported bilateral TKAs with concomitant femoral osteotomies [7]. However, no published reports describe simultaneous bilateral TKA in achondroplasia. Recent literature suggests that there are no differences in clinical outcomes between simultaneous and staged bilateral TKA procedures, and simultaneous TKA may be more appropriate in the setting of symptomatic bilateral end-stage OA [8–10]. Despite the technical challenges of TKA in SD, favorable outcomes and survivorship have been reported [11].

We present a unique case of a patient with achondroplasia who underwent one-stage bilateral hinged knee replacements for extreme varus knee deformities over 40 degrees and discuss the preoperative challenges, intraoperative strategies, implant considerations, and outcomes in comparison with the existing literature. The patient was informed that data concerning the case would be submitted for publication and provided consent.

## Case report

**Figure 1:**
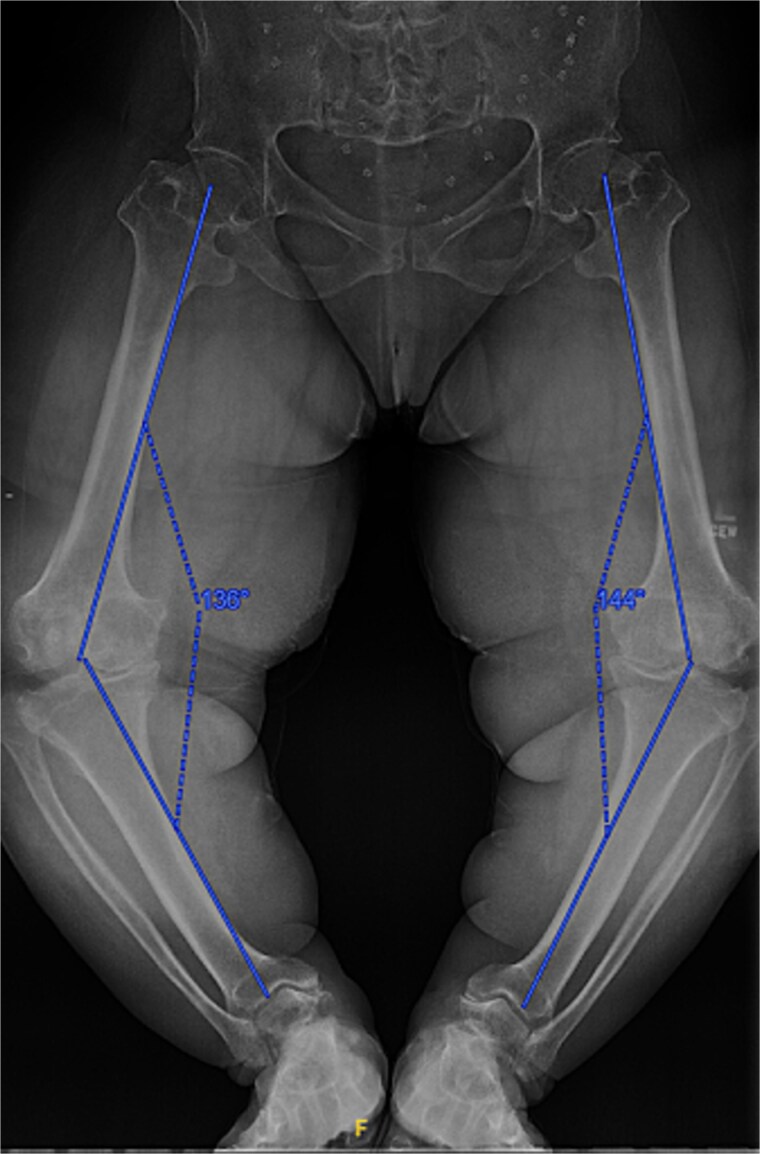
Preoperative standing AP radiographs demonstrating severe bilateral mechanical axis varus deformity measuring 36 and 44 degrees on the left and right legs, respectively.

A 61-year-old woman with a history of achondroplasia, gastroesophageal reflux disease, depression, and sciatica presented with progressively worsening bilateral knee pain, mechanical symptoms, and significant difficulty with ambulation. The patient had a body mass index (BMI) of 41.1 kg/m^2^ and a characteristic short-limb SD phenotype consistent with achondroplasia. On physical examination, the patient demonstrated bilateral varus alignment of the lower extremities and gait assessment revealed a significant varus thrust and valgus instability in mid-stance. Bone length radiographs revealed severe medial joint space narrowing, metaphyseal flaring of the tibia and femur, and bilateral mechanical axis varus deformity measuring 36 and 44 degrees on the left and right legs, respectively (Fig. 1).

**Figure 2:**
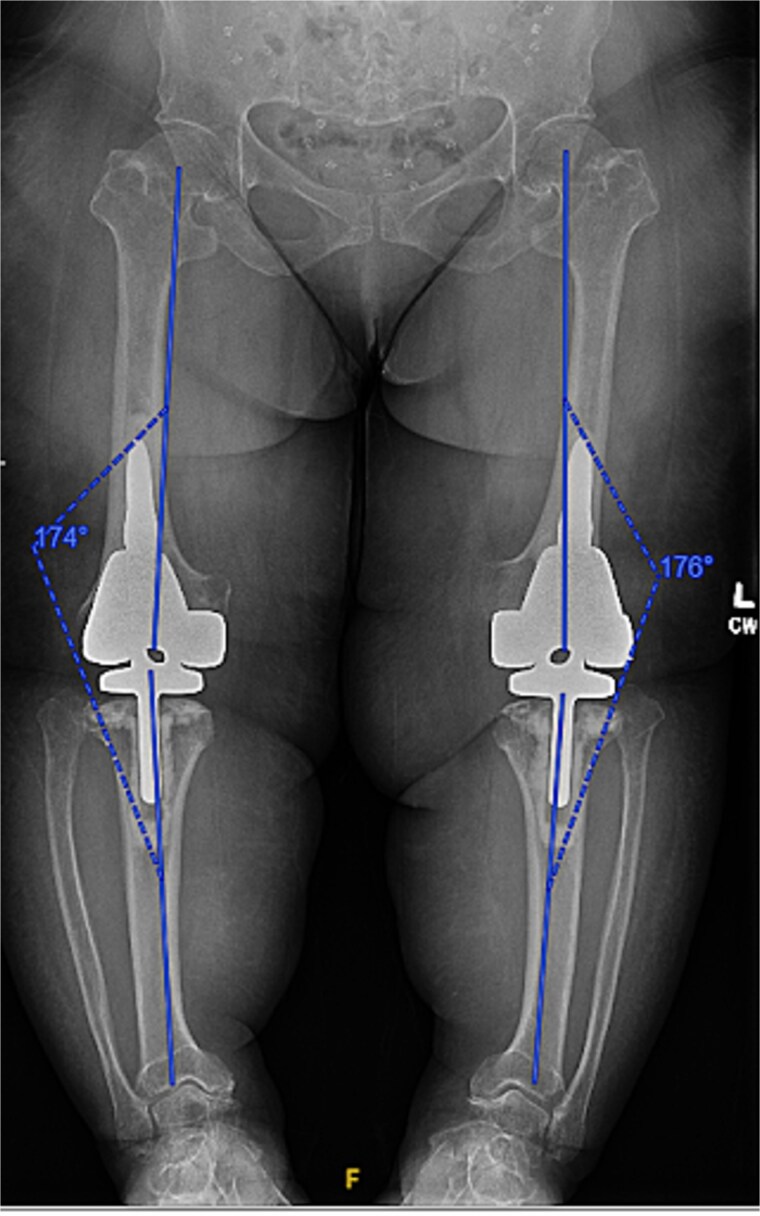
Immediate postoperative AP radiographs following bilateral robot-assisted rotating-hinge TKA, showing well-aligned prosthetic components and correction of the coronal deformity. The post-operative mechanical axis for the left and right knees were 4 and 6 degrees, respectively.

Given the severity of the coronal deformity, ligamentous laxity, and the patient’s functional limitations, the decision was made to proceed with simultaneous bilateral robot-assisted TKA using constrained prostheses. Using robotic-assisted navigation (Stryker Mako™), bony landmarks and soft tissue gaps were registered on the right knee. Initial preparation for a posterior-stabilized implant demonstrated profound lateral laxity after medial releases, prompting intraoperative conversion to a rotating-hinge design. A size XS femoral component and pediatric all-polyethylene tibial component (8 mm) were cemented, followed by hinge assembly. A similar approach was used for the left knee as severe medial compartment collapse and lateral laxity again necessitated the use of a rotating-hinge construct. The femur was prepared for an extra-small femoral component with a 12 × 50 mm cemented stem, and a size 8 mm pediatric all-polyethylene tibial component was implanted. The post-operative mechanical axis for the left and right knees were 4 and 6 degrees, respectively (Fig. 2).

**Figure 3:**
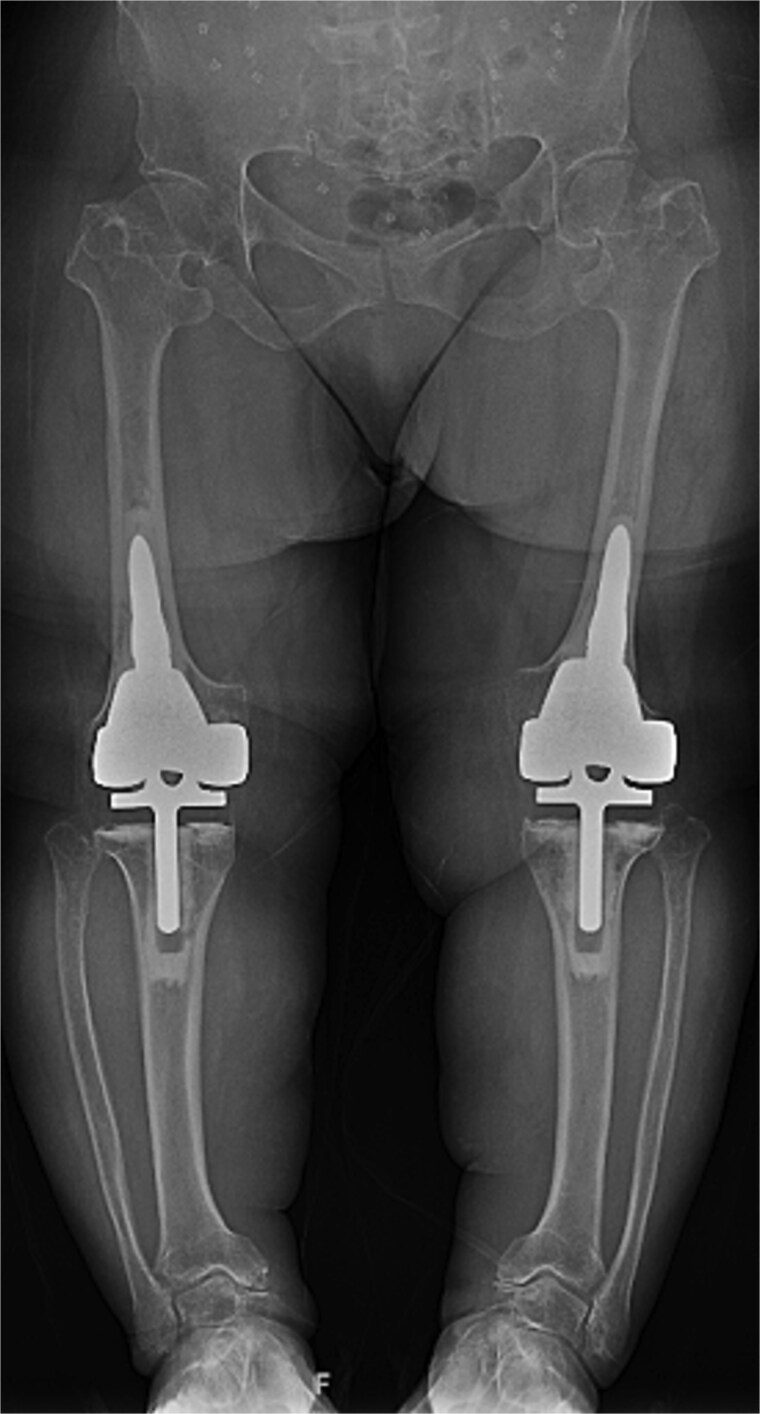
Two-year postoperative AP radiographs demonstrating stable, well-fixed components with appropriate alignment and no evidence of implant loosening or periprosthetic lucency.

**Figure 4:**
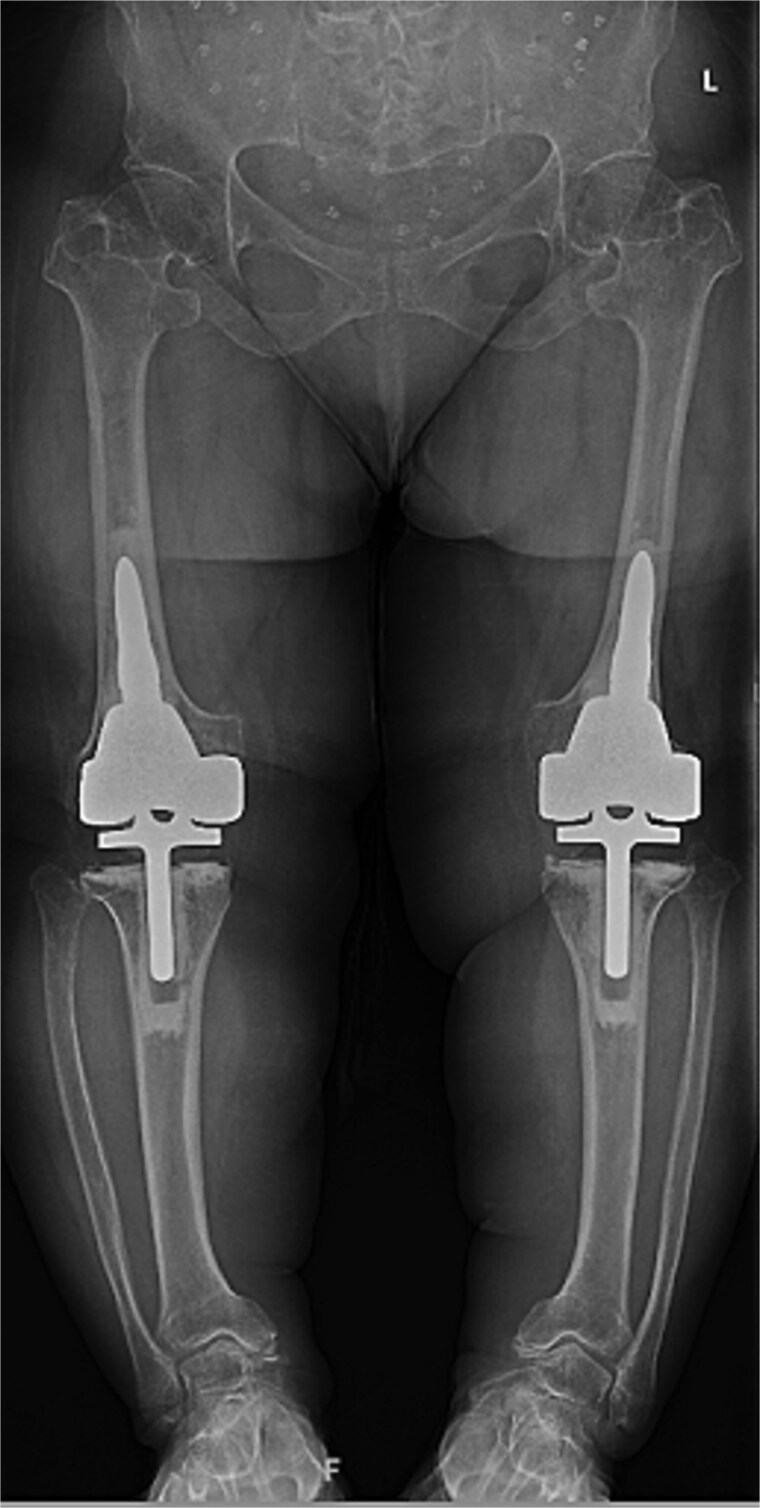
Five-year postoperative AP radiographs showing continued stability of bilateral rotating-hinge implants with maintained alignment and no radiographic evidence of implant failure.

At the 2-year follow-up, the patient reported no significant pain, demonstrated a normal gait, and had active bilateral knee range of motion from 0° to 125°. There was no joint line tenderness or instability. Surveillance radiographs confirmed well-aligned and stable components with no evidence of implant failure or periprosthetic lucency (Fig. 3). At the five-year follow-up, the patient remained very satisfied with her knees and denied knee pain. On exam, her gait was slightly antalgic, but active bilateral knee range of motion remained preserved at 0° to 120°, and both knees remained stable throughout range of motion and nontender to palpation. Radiographs continued to show stable prosthetic components without evidence of implant failure or periprosthetic lucency (Fig. 4).

## Discussion

Achondroplasia, caused by a FGFR3 mutation, results in rhizomelic shortening, metaphyseal flaring, and characteristic genu varum from both extra-articular bowing and intra-articular degeneration [1, 2]. These anatomic features complicate conventional arthroplasty techniques, requiring careful preoperative planning, templating, and component selection [3].

Previous reports have detailed the management of severe deformity in patients with achondroplasia using both staged TKA and combined osteotomy approaches. Walter *et al*. reported a staged bilateral TKA with concomitant femoral osteotomy for correction [7], while Bayle-Iniguez *et al*. maintained slight varus alignment without osteotomy, achieving satisfactory 11-year results [12]. In our patient, full correction was achieved intra-articularly using robotic assistance and constrained implants, avoiding osteotomies while restoring mechanical alignment.

Implant selection is critical in SD due to ligamentous laxity and bony mismatch. Rotating-hinge designs have proven effective in the setting of significant instability, as previously reported by Stancil *et al*. [6] and Ali *et al*., who found over half of dysplasia-related TKAs required hinged prostheses [11]. Downsizing to pediatric all-polyethylene tibial inserts (8 mm) was necessary in our case to avoid cortical compromise. The frequent need for custom components in skeletal dysplasia is well-documented, with custom implants or jigs required in 38.7% and 19.4% of cases, respectively, in one series [11]. Surgeons must anticipate these challenges preoperatively and coordinate with manufacturers as needed [3].

Research evaluating the long-term outcomes in achondroplasia remains limited. A recent systematic review reported 92% survivorship at 10 years, decreasing to 46% at 20 years in patients with skeletal dysplasia [13]. Simultaneous bilateral TKA, though associated with higher surgical complication rates, may reduce overall medical morbidity and costs when compared to staged procedures [8–10]. Infection risk appears elevated in patients with SD compared to patients without SD (*P* = .044) [14], warranting vigilant aseptic technique and perioperative protocols. Neurologic complications, such as peroneal nerve palsy, are more often associated with valgus correction, but nerve stretch injuries remain a risk with any large deformity correction [3, 15].

## Conclusion

This is one of the first reported cases of simultaneous bilateral TKA in a patient with achondroplasia and extreme bilateral varus deformity, highlighting how successful correction can be achieved using hinged implants and careful surgical planning, underscoring the feasibility of one-stage bilateral intervention in patients with achondroplasia.
